# Introgression of a *cry1Ab* transgene into open pollinated maize and its effect on Cry protein concentration and target pest survival

**DOI:** 10.1371/journal.pone.0226476

**Published:** 2019-12-16

**Authors:** Reynardt Erasmus, Rialet Pieters, Hannalene Du Plessis, Angelika Hilbeck, Miluse Trtikova, Annemie Erasmus, Johnnie Van den Berg

**Affiliations:** 1 Unit for Environmental Sciences and Management, North-West University, Potchefstroom, South Africa; 2 ETH Zurich, IBZ, Plant Ecological Genetics, Zurich, Switzerland; 3 ARC-Grain Crops, Potchefstroom, South Africa; University of Texas at Dallas, UNITED STATES

## Abstract

In Africa, the target pests of genetically modified Bt maize are lepidopteran stem borers, notably *Busseola fusca* (Lepidoptera: Noctuidae). Gene flow between Bt maize hybrids and open pollinated varieties (OPVs) that do not contain the Bt trait is highly likely in areas where both types of maize are cultivated. Consequently, introgression of the *cry1Ab* transgene into local OPVs will result in unknown patterns of Cry1Ab protein expression in plants during follow-up seasons when recycled seed of OPVs is planted. Too low concentrations of Cry protein in such plants may result in selection for resistant alleles and accelerate resistance evolution. The aim of this study was to determine the effects of introgression of the *cry1Ab* transgene into an OPV, on Cry protein concentration levels and pest survival. Bt transgene introgression was done by crossing a transgenic donor hybrid containing the *cry1Ab* gene with a non-Bt OPV as well as with a non-Bt near-isogenic hybrid. F1 and F2 crosses as well as back crosses were done yielding 11 genotypes (treatments). Cry1Ab protein concentrations in leaf tissue of these crosses were determined by means of ELISAs. All crosses that contained the transgene had similar or higher Cry1Ab concentrations when compared to the Bt parental hybrid, except for the Bt x OPV F1-cross that had a significantly lower Cry1Ab concentration. Survival *B*. *fusca* larvae were evaluated in assays in which larvae were reared for 14 days on whorl leaf tissue of the different treatments. Larval survival did not differ between any of the maize plant treatments which contained the Bt gene. Results suggest that Bt transgene introgression into OPVs may produce plant progenies that express Cry1Ab protein at sufficient concentrations, at last up to the F2 seed, to control *B*. *fusca* larvae. Resistance evolution is however not only influenced by the frequency of pest individuals that survive exposure to the Cry proteins but also by factors such as genetics of the pest and recipient OPV, pest biology and migration behaviour.

## Introduction

Since the first deployment of genetically modified Bt crops concerns were raised about resistance evolution in target pest species [1,2]. After more than 20 years of Bt crop cultivation, there are now 39 confirmed cases of resistance in 15 pest species worldwide, nine of which belong to the Noctuidae (Lepidoptera) family [3–8].

The rates at which pests develop resistance to Bt toxins can be exacerbated by increased larval migration, low-dose expression and non-compliance to refuge requirements [9–12]. Insect resistance management (IRM) in Africa is faced by several challenges such as recycling of seed, gene flow between varieties and small units of land. While large scale industrial and small scale subsistence farming is done in close proximity in some countries, in others, many small fields cultivated by different farmers are situated in close proximity, which allows for gene flow to occur between maize varieties [13,14]. Contamination can occur in many ways. Either Bt seed, non-Bt seed, or both can be contaminated by seed mixing or gene flow and introgression [15,16]. Low dose expression of Cry toxins is a challenge to IRM, especially in subsistence farming where crop fields are small, Bt maize OPV’s may be cultivated in adjacent fields, and seed is recycled. There are no genetic barriers that prevent gene flow between landraces and Bt hybrids [17,18]. Gene flow in environments where Bt maize and OPVs co-exist will result in introgression of transgenes into OPV’s [13,14,19,20], providing challenges to IRM in the African context [17,21,22].

To counter resistance evolution in target pests of Bt maize, farmers need to employ specific IRM practices [23–25]. These IRM strategies are based on cultivation practices in large-scale systems [17]. The high-dose/refuge strategy is an example of such an IRM practice originally designed for application in large-scale commercial farming systems [2]. IRM strategies, especially planting of structured non-Bt maize refuges, may be difficult to implement in a small holder farmer context [26–28] and implementation thereof provides a huge challenge to small farmers and subsequently to stewardship of the technology in small holder farming systems [29,30].

African small holder farming practices and landscape features are not conducive to the application of good stewardship practices, such as those applied in industrial farming systems. Small holder fields are small and in close vicinity to fields of neighbouring farmers, maize seeds are often recycled after the season and shared amongst farmers and different varieties of maize are often planted together in a single field [13,17,22,31]. These practices will contribute to the flow of transgenes into OPVs. An implication of such events is that unknown patterns of Bt expression may occur within farmer’s fields planted with a mix of progeny from recycled seeds, some expressing Cry proteins at unknown and likely variable concentrations. This in turn has unknown effects on the evolution of resistance in target pest species in maize production systems [32]. Chilcutt and Tabashnik [33] suggested that transgene flow into non-Bt maize hybrids would accelerate resistance evolution by selecting for heterozygotes. Varying Cry protein levels within plants [21,34] and low-dose expression [35] have also been reported to largely contribute to resistance evolution.

Krupke et al. [36] suggested that gene flow would result in F1 volunteer maize plants with decreased fitness and lower efficacy at controlling the western corn rootworm, *Diabrotica virgifera virgifera* LeConte (Coleoptera: Chrysomelidae). On the other hand, introgressed OPVs expressing *cry* genes could also increase the fitness of plants by protecting them when they are subjected to target pest attack. In such cases, farmers that recycle seed may unknowingly select maize ears and seeds with these favourable traits containing the Bt transgene and save them for planting during the next season. Transgene frequency in small holder farming systems may, therefore, increase within local landraces and OPVs [13,14,17].

Extensive and repeated gene flow from GM to non-GM plants, or to already introgressed GM OPVs (back crossing) via pollen dispersal may lead to potentially wide ranging heterogeneity of these traits over time. This may result in highly variable expression levels of transgenes and Cry protein concentrations. Bt toxin concentration in plant tissue following the introgression of the Bt transgene into conventional non-Bt maize hybrids has been reported to decline after the second generation of backcrosses to a non-GM hybrid [37]. The indirect implications of such altered protein expression levels have been reported to support the evolution of resistance to Cry proteins in target pest species [32,38] and may lead to reduced effectiveness of the high-dose/refuge strategy that is employed as IRM strategy. Therefore, if Cry protein concentrations in OPVs are too low to kill sufficient numbers of the target pest, resistance may develop largely unnoticed in small-scale farming systems, following the introgression of Bt transgenes into OPVs. Little information exists on the effects of Bt transgene flow to OPV maize, except for that of Fearing et al. [39], who indicated that the *cry1Ab* gene was stable over four generations of backcrosses in two different Bt maize lines. Glaum et al. [21] reported that transgene flow from Bt maize to non-Bt maize in refugia would accelerate the rate of insect resistance evolution and could potentially compromise IRM.

The aim of this study was to determine the effects of introgression of the Bt *cry1Ab* transgene into an open pollinated maize variety on Cry protein concentrations and the subsequent survival of the most important target pest, *B*. *fusca*, on such plant crosses. Results of this study will contribute to development of appropriate IRM strategies in Africa.

## Material and methods

### Crosses to obtain experimental material

**Fig 1:**
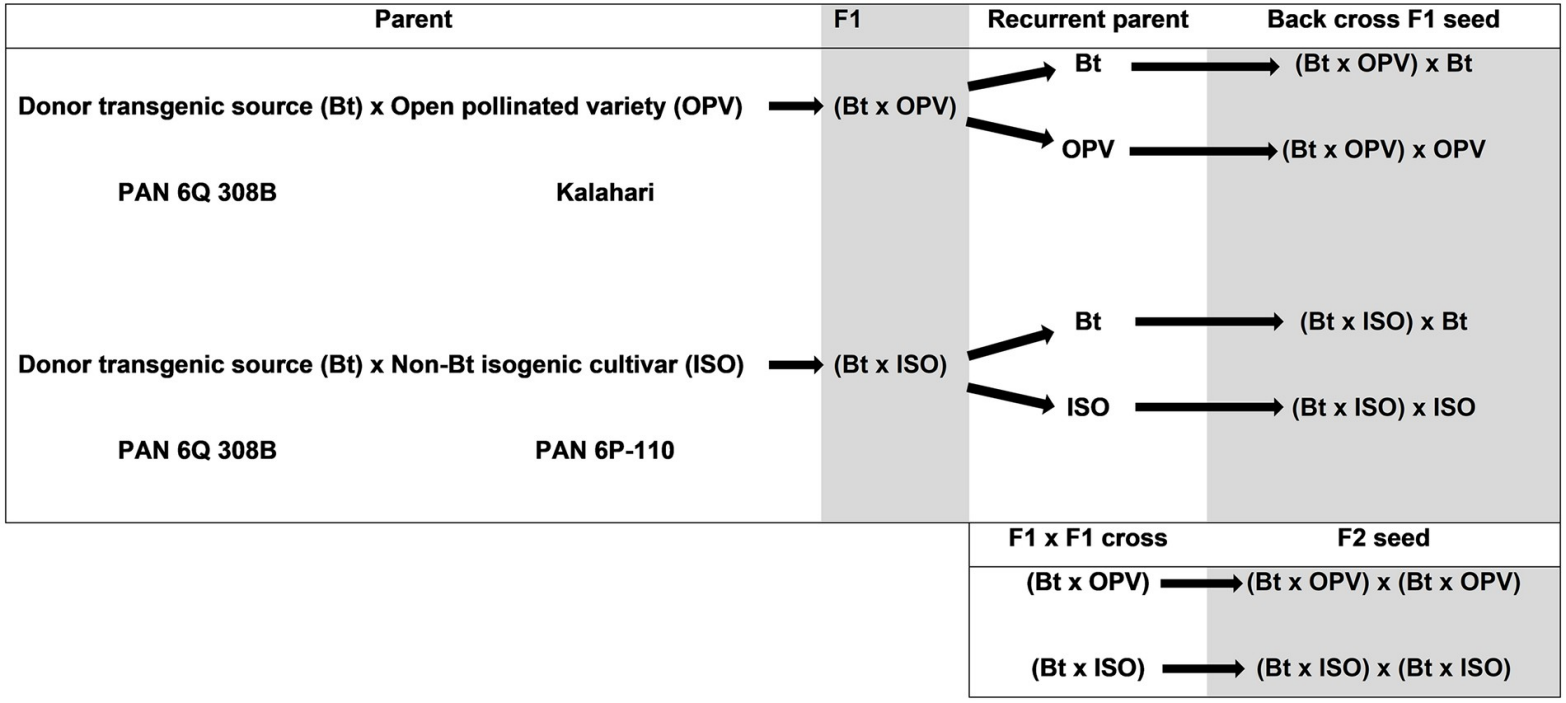
Diagram indicating the process of transgene introgression. Crosses (F1 crosses, F2 crosses and backcrosses) made between the transgenic donor source (Bt maize), an open pollinated variety and a non-Bt maize cultivar to obtain experimental material.

Bt transgene introgression was done by crossing an OPV (Kalahari) with a transgenic donor hybrid (Bt maize: PAN 6Q 308B), containing the *cry1Ab* gene that codes for expression of Cry1Ab protein. A diagram illustrating the crosses that were made is provided in Fig 1. The Bt hybrid was also crossed with the non-Bt hybrid (PAN 6P-110 = ISO) which is the near-isogenic hybrid to PAN 6Q-308B. F1 seed from the crosses Bt-OPV and Bt-ISO were produced and harvested. These F1 seeds were planted and the presence of the Cry1Ab protein in seedlings was confirmed by means of ENVIROLOGIX^®^ test strips. Seedlings that did not test positive for the Cry1Ab protein were removed from the trial plots. Plants that tested positive for the Cry1Ab protein were grown to maturity and during flowering, back crosses were made with all three recurrent parents (Bt, isogenic hybird and OPV) as well as F2 crosses (Bt-OPV x Bt-OPV and Bt-ISO x Bt-ISO) (Fig 1). Plants of all back crosses, as well as seedlings of F1 and F2 seed, were also tested by means of ENVIROLOGIX^®^ test strips to confirm the presence of the Cry1Ab protein prior to conducting the experiments with *B*. *fusca*.

### *Busseola fusca* stock colony

A *B*. *fusca* population was sampled from the Venda region (23°3’S; 30°3’E) in the Limpopo Province of South Africa, where small holder famers do not plant Bt maize and where this pest is still highly susceptible to the Cry1Ab protein [40]. Larvae were collected from freshly cut non-Bt maize stems. Collected larvae were reared in plastic containers (40 x 20 x 15 cm) with aerated lids on non-Bt maize stems which were replaced at four-day intervals in a rearing chamber at 26 ± 1 °C, 70 ± 5% RH and 14L: 10D photoperiod, until pupation. The F1-generation offspring of this field-collected population was used in the bioassays.

### Bioassays

Survival of *B*. *fusca* larvae was evaluated on the Bt hybrid, its non-Bt isogenic hybrid, the OPV and the eight subsequent maize crosses described above (Table 1). Each of these 11 treatments was replicated three times. Each replicate consisted of 10 plastic containers (52 mm high and 30 mm in diameter) with five neonate larvae per container. *Busseola fusca* larvae are not cannibalistic and feed in confined plant whorl at high larval densities for a period of approximately 14 days before the bore into maize stems [9]. Whorl leaf tissue from a single maize plant was provided as food to the larvae in each single container. Plants were in the V6–V8 growth stages.

**Table 1:** Cry1Ab protein concentrations and percentage survival of *Busseola fusca* larvae. Mean Cry1Ab protein concentrations (μg/g dry weight) in leaf tissue of different maize treatments, descriptive statistics of Cry1Ab protein concentration such as the median, first quartile (q1), third quartile (q3) and coefficient of variation (CV%), and mean percentage survival of *Busseola fusca* larvae after 7 and 14 days of feeding on the respective treatments and Spearman rank-order correlations (rs) between Cry1Ab protein concentration and percentage larval survival after seven days.

	Mean concentration	Median (q1; q3)	Number of plants	CV%	Survival (%)		
Maize treatments	Cry1Ab (μg/g)				7 days	14 days	(rs)
Bt (Parental line)	14.9 ± 1.3^b^	13.7 (12.5; 16.9)	29	29.5	9.3 ± 3.4^a^	6.0 ± 3.0^a^	0.08
OPV (Parental line)	-	-	30	-	77.3 ± 3.4^b^	53.3 ± 3.0^b^	-
ISO (Parental line)	-	-	30	-	62.7 ± 3.4^b^	46.7 ± 3.0^b^	-
Bt x OPV	8.9 ± 1.4^c^	8.7 (6.8; 10.4)	26	34.5	24.0 ± 3.8^a^	13.6 ± 3.3^a^	-0.06
Bt x ISO	16.6 ± 1.3^ab^	15.8 (13.7; 20.3)	29	29.2	16.2 ± 3.7^a^	10.0 ± 3.2^a^	0.09
(Bt x OPV) x Bt	18.3 ± 1.4^ab^	16.2 (9.7; 20.9)	25	62.3	19.1 ± 4.0^a^	10.9 ± 3.5^a^	-0.002
(Bt x OPV) x OPV	14.9 ± 1.3^b^	15.4 (11.8; 17.6)	30	34.4	17.1 ± 3.6^a^	11.4 ± 3.1^a^	0.02
(Bt x ISO) x Bt	24.0 ± 1.3^a^	23 (18.1; 29.2)	29	34.5	16.2 ± 3.7^a^	14.6 ± 3.2^a^	0.15
(Bt x ISO) x ISO	18.9 ± 1.3^ab^	17.9 (15.4; 22.1)	28	28.5	16.4 ± 3.6^a^	8.6 ± 3.1^a^	-0.08
(Bt x OPV) x (Bt x OPV)	19.7 ± 1.3^ab^	19.2 (14.6; 22.5)	27	32.1	19.3 ± 3.6^a^	9.3 ± 3.1^a^	-0.25
(Bt x ISO) x (Bt x ISO)	23.1 ± 1.3^a^	22.3 (15.9; 30.0)	29	41.5	14.7 ± 3.4^a^	5.3 ± 3.0^a^	-0.08
	H(10) = 132.37				H(8) = 82.91	H(10) = 118.02	

Different superscripts in a column denote significant differences (Kruskal-Wallis followed by Dunn’s multiple comparison test; p<0.05)

Fresh leaf material was provided to larvae at four-day intervals. Plant tissue of 15 plants of each of the respective maize treatments were used as food for larvae on each occasion. Whorl tissue (2 x 5 cm) of a single plant was used as food for larvae in only two replicates. This was done ensure that a large number of plants (15) of each treatment was used in this study and to ensure that larvae were exposed to to a representative range of concentrations of Cry protein in the particular maize treatment.

Larvae were kept in the containers under similar conditions as those described above for the stock colony. Larval survival was recorded at days seven and 14. The experiment was terminated after 14 days when larvae were in the 3^rd^ instar. This 14-day period was used since previous studies with *B*. *fusca* on Bt maize indicated that Cry proteins kill susceptible larvae within 12 days [40–42].

A small piece (approximately 2 x 2 cm) of the central whorl leaf of each plant was sampled sampled immediately before the rest was provided as food to larvae. These leaf samples were frozen in liquid nitrogen immediately after sampling and stored at -80 °C until enzyme-linked immuno-sorbent assays (ELISAs) were done to determine the Cry1Ab protein concentration in each sample.

### ELISA

ELISA data can be quantified when interpreted in association with a standard curve (a set of serial dilutions of a known antigen). In order to adapt the ELISA kit for quantitative purposes, Cry1Ab analytical standard reference protein was acquired from Marianne P. Carey, Case Western Reserve University, School of Medicine, Cleveland, USA. These proteins were purified by high pressure liquid chromatography (HPLC) from salt-free *Bacillus thuringiensis* full size proteins.

The frozen maize whorl leaf samples were freeze dried and 6–10 mg of each sample of leaf material was used for the ELISAs. The mass of each sample was determined before grinding the sample into a fine powder, using a Retch bead rupture. Two steal beads were added per sample and the bead rupture placed on a 30 m/s setting for 3 minutes. The Cry1Ab protein was extracted from the leaf powder by adding 1.5 ml phosphate buffered saline Tween-20 (PBST) buffer (pH 7.4). After centrifugation, the supernatants were all diluted 1:100 with PBST-buffer. The Cry protein levels of the sampled whorl material were determined by using antibody coated wells of Envirologix^®^ (qualiplate combo kit AP 045) for Cry1Ab. Standards of Cry1Ab at concentrations of 0.03, 0.06, 0.12, 0.24, 0.5, 1, 1.5, 2, 2.5, 3 and 3.5 ng/mℓ were used to construct standardised optical density curves for estimating the Cry1Ab protein content of samples. All standards and samples were added in duplicate to a 96-well ELISA plate. Two standard curves were run per analysis. A positive control included in the Envirologix^®^ kit, was added in duplicate to the plate. The colour intensity was measured at 450 nm with a reference wavelength of 650 nm by using a microplate reader (Berthold TriStar LB941). Sample Cry1Ab protein content was calculated by using the linear regression equation for the standard curve. These concentrations were then back calculated to account for mass. Cry1Ab concentrations in the whorl samples were expressed as μg/g dry mass. Non-Bt plant material were also analysed to confirm the absence of Cry1Ab protein from these samples and to ensure that there was no cross-reaction of the antibodies with other plant compounds.

### Data analyses

Data for larval survival and Cry1Ab concentration were analysed for normality with the Shapiro–Wilk test, and for homogeneity, the Levene’s test was used. Data were neither normally distributed nor homoscedastic, therefore the data were analysed using the Kruskal–Wallis test.

All 11 maize treatments (parental lines as well as Bt maize plant crosses) were included in the analyses of larval survival but due to the absence of Bt protein in the non-Bt OPV and non-Bt isogenic hybrid treatments, these two were excluded from the analyses. Correlations between Cry1Ab expression levels in plant tissues and percentage larval survival were analysed using Spearman’s rank-order correlation. All statistical analyses were performed using STATISTICA 13 (Statsoft Inc., 2013).

## Results

### Cry1Ab concentrations in different maize crosses

The Bt x OPV F1-cross had the lowest Cry1Ab concentration (8.9 μg/g) (Table 1) and differed significantly from the Bt parental hybrid as well as all other maize crosses. Plants of the (Bt x ISO) x Bt backcross had the highest mean Cry1Ab concentration (24 μg/g) and together with the (Bt x ISO) x (Bt x ISO) F2-cross (23.1 μg/g) (H(8) = 82.91; p < 0.001), differed significantly from the Bt parental hybrid with regards to Cry1Ab protein concentration.

There was a large variation in Cry1Ab concentration in the Bt hybrid as well as Bt maize crosses with coefficients of variance (CV) ranging from 28.5 to 62.3% (Table 1). Data regarding Cry1Ab concentration in Bt maize plant crosses had a CV approximately equal to or higher than that of Bt maize plants. The variation in Cry1Ab protein concentration levels within each maize treatment was also evident from the median (data point at 50^th^ percentile) and quartile 1 (data point at 25^th^ percentile) and quantile 3 (data point at 75^th^ percentile) values provided in Table 1.

### Survival of *B*. *fusca* larvae on different maize crosses

Larval survival after seven days of feeding on the OPV and the iso-line was 77.3 and 62.7% respectively (Table 1). Larval survival was similarly high on these two parental lines after 14 days of larval feeding, with 53.3 and 46.7% respectively, which differed significantly from all Cry1Ab containing maize treatments. Survival of *B*. *fusca* larvae on the various Bt-maize crosses was much lower than that on non- Bt treatments and ranged between 14.7 and 24.0% after 7 days and 5.3 and 14.6% after 14 days. No significant differences were observed in survival between larvae that fed on the respective Cry1Ab containing maize treatments after both seven (H(10) = 132.37; p<0.001) and 14 days (H(10) = 118.02; p<0.001) of feeding. No significant correlations were observed between the measured Cry1Ab toxin concentration in Bt maize tissue and larval survival (Table 1).

## Discussion

Introgression of transgenes into other maize varieties can occur in many ways and, due seed sharing and recycling, this could be a common occurrence in African small holder farming systems [13,15,16,31].

Stable inheritance and expression of transgenes in Bt plants are vital for the efficacy of Bt crops [43,44]. The stability of transgene introgression and expression has been widely studied in a variety of GM crop species. Previous studies indicated that transgenes are inherited as a dominant trait with inheritance conforming to a 3:1 Mendelian ratio [39,45–51].

In this study the Cry1Ab concentration in the parental Bt hybrids did not differ notably from that of most other treatments, despite the high levels of variation in Cry1Ab concentrations within some crosses. High variation in the Bt protein concentration was observed in all the crosses. Similar high levels of variation in maize crosses were reported by Trtikova et al. [52]. According to Xia et al. [50] large variation in the Bt protein content occurs in transgenic Bt rice plants. They also reported that the Bt transgene expressed as expected in interspecific hybrids between genetically modified rice and common wild rice and that the the Cry protein was still highly active against target insects.

The high concentrations of Cry1Ab protein in some plants produced by crosses in this study could probably be ascribed to carrying the Bt gene on both alleles. Commercially available Bt maize hybrids are hemizygous, compared to the backcrosses in this study, which was done with Bt-containing maize, resulting in half of the progeny being homozygous for the Bt transgene. It is therefore possible that the high Cry1Ab protein expressing plants of the (Bt x ISO) x Bt back crosses might have carried the Bt transgene on both alleles, while lower expression can be expected from plants containing only one allele of the transgene. The same applies to plants of the (Bt x ISO) x (Bt x ISO) F2-cross. Higher concentrations were prominent in plants produced by the (Bt x OPV) x Bt cross. The mean Cry1Ab concentration levels in the latter were higher than the median which indicates that the recorded variation in Cry1Ab expression was skewed towards the higher concentration levels.

The Cry1Ab concentrations in none of the crosses in this study were significantly lower than that in plants of the parental Bt maize hybrid, except for the Bt-OPV F1 cross. The general similarity of the mean Cry1Ab concentrations and larval survival on plant tissues of the respective treatments in this study indicate transgene stability over generations, similar to a report by Fearing et al. [39], who indicated stability of the cry*1Ab* gene over four generations of maize backcrosses. Micallef et al. [49] also reported stable expression of transgenes in Alfalfa, while Wang et al. [53] reported high expression of the Bt gene in hybrid Bt rice plants derived from crosses between conventional rice varieties and Bt transgenic rice.

The low survival of *B*. *fusca* larvae on the various Bt-maize crosses show that the outcrossed cry1Ab transgene remained active in all crosses where it was present, although some larvae survived in each of the treatments (no zero survival). The relatively low levels of larval survival (53.3 and 46.7%) on the two non-Bt maize treatments are typical for *B*. *fusca*, which is notoriously difficult to rear under artificial conditions. Strydom et al. [40], Khan et al. [54] and Van den Berg et al. [55] reported similar levels of survival on plants grown in pots in greenhouses.

While non-Bt near-isogenic genotypes are closely related to their GM counterparts, OPV maize plants are highly variable with open evolving genetic systems [56]. The genetic diversity of local maize varieties and landraces are unknown in most areas of the world [17]. Since transgene expression in maize is affected by genetic background [57] the lower Cry1Ab protein concentration levels observed in these crosses made with the OPV can possibly be ascribed to the large differences in genetic background between the Bt parental hybrid and the OPV (Kalahari) used to produce crosses. Xia et al. [50] and Bakó et al. [58] also ascribed the large variation in transgene expression in crosses between Bt and common wild rice, and crosses between non-GM maize inbred lines and a Cry3Bb1 maize line to genetic background effects.

Results indicated that individuals of the recipient maize population benefited from the insecticidal properties of the Bt transgene by providing protection against borer damage. In small holder farming systems where the healthiest maize ears and seed are selected for the next years’ crop, this may indirectly favour farmer selection behaviour for seeds from undamaged plants, thereby possibly accelerating the rate of transgene introgression. Such selection pressure on the recipient population by farmers selecting seed to plant the following year was reported by Bellon and Berthaud [59]. Seed selection and mixing as well as pollen transfer between plants and fields are affected by the features inherent in the maize grown, agroecological circumstances and farmer’s practices [21]. Modelling of resistance evolution of pests following gene flow from Bt maize fields to non-Bt maize refuges indicated that refuge contamination had generally small effects on on the rate of evolution [21]. Based on the latter study, the authors also concluded that transgene introgression resulting from saving of such seed is unlikely to have a major effect on resistance evolution [21]. Even if scenarios such as those discussed above lead to the increased rate of transgene introgression into OPVs, the results of this study indicate a stable expression of the transgene up to F2 seed which should have no major effect on resistance evolution.

## Conclusions

The evolution of resistance to Cry proteins in target pests is a major threat to the continued successful use of Bt crops [60,61], emphasizing the importance of knowing the effects of varying Cry protein concentrations in OPVs and Bt-introgressed non-Bt hybrids in uncontrolled environments. This study demonstrated the effects of introgression of a Bt transgene into a non-Bt OPV and on the subsequent concentration of the Cry1Ab protein. The Cry1Ab protein concentrations varied largely within Bt maize plant crosses, but the concentration of the Cry1Ab protein within the respective crosses were never significantly lower than that of the parental Bt hybrid. In many cases the Cry1Ab concentrations were higher in crosses than in the Bt parent, even at the F2 level. This study only focused on the effects of introgression of the *cry1Ab* gene on Cry1Ab concentration levels in F1 crosses, backcrosses and F2 crosses with a single OPV. The introgression effects in later generations and possible effect of the genetic background of the OPV are therefore yet unknown. Based on the high Cry1Ab protein content in plants after successive introgressions in this study, as well as reports [21;33] that gene flow from Bt hybrids to non-Bt maize refuge plants have little effect on the rate of resistance evolution, we conclude that concerns regarding influence of gene flow from Bt maize to OPVs in African smallholder farming systems may be overestimated.

## Supporting information

S1 Table Cry1Ab protein concentration data of different maize treatments. (DOCX)

S2 Table Larval mortality data on the different maize treatments. (DOCX)
