# Supplementary material for: Introgression of a cry1Ab transgene into open pollinated maize and its effect on Cry protein concentration and target pest survival
Source: PLoS One. 2019 Dec 16;14(12):e0226476. doi: 10.1371/journal.pone.0226476 (PMC6914330; doi:10.1371/journal.pone.0226476)
Supplement: S1 Table — (DOCX) [file pone.0226476.s001.docx]

**Table S1**

| **Maize treatment** | **Plant number** | **Cry1Ab protein concentration (µg/g)** |
| --- | --- | --- |
| Bt | 1 | 25.33 |
| Bt | 2 | 16.16 |
| Bt | 3 | 20.06 |
| Bt | 4 | 15.54 |
| Bt | 5 | 9.29 |
| Bt | 6 | 15.65 |
| Bt | 7 | 13.04 |
| Bt | 8 | 9.94 |
| Bt | 9 | 10.03 |
| Bt | 10 | 13.89 |
| Bt | 11 | 17.53 |
| Bt | 12 | 21.79 |
| Bt | 13 | 13.73 |
| Bt | 14 | 27.11 |
| Bt | 15 | 16.93 |
| Bt | 16 | 10.52 |
| Bt | 17 | 17.79 |
| Bt | 18 | 13.36 |
| Bt | 19 | 9.53 |
| Bt | 20 | 13.29 |
| Bt | 21 | 13.49 |
| Bt | 22 | 12.08 |
| Bt | 23 | 14.25 |
| Bt | 24 | 12.57 |
| Bt | 25 | 12.48 |
| Bt | 26 | 17.62 |
| Bt | 27 | 15.27 |
| Bt | 28 | 13.35 |
| Bt | 29 | 10.84 |
| Bt x OPV | 1 | 13.25 |
| Bt x OPV | 2 | 11.61 |
| Bt x OPV | 3 | 10.09 |
| Bt x OPV | 4 | 12.42 |
| Bt x OPV | 5 | 10.06 |
| Bt x OPV | 6 | 8.89 |
| Bt x OPV | 7 | 11.53 |
| Bt x OPV | 8 | 4.62 |
| Bt x OPV | 9 | 6.78 |
| Bt x OPV | 10 | 15.45 |
| Bt x OPV | 11 | 3.31 |
| Bt x OPV | 12 | 3.48 |
| Bt x OPV | 13 | 6.78 |
| Bt x OPV | 14 | 8.44 |
| Bt x OPV | 15 | 9.88 |
| Bt x OPV | 16 | 10.37 |
| Bt x OPV | 17 | 9.25 |
| Bt x OPV | 18 | 13.95 |
| Bt x OPV | 19 | 10.22 |
| Bt x OPV | 20 | 6.04 |
| Bt x OPV | 21 | 6.31 |
| Bt x OPV | 22 | 7.89 |
| Bt x OPV | 23 | 7.73 |
| Bt x OPV | 24 | 6.52 |
| Bt x OPV | 25 | 7.52 |
| Bt x OPV | 26 | 8.59 |
| Bt x ISO | 1 | 20.37 |
| Bt x ISO | 2 | 21.81 |
| Bt x ISO | 3 | 14.99 |
| Bt x ISO | 4 | 11.72 |
| Bt x ISO | 5 | 20.69 |
| Bt x ISO | 6 | 22.39 |
| Bt x ISO | 7 | 20.30 |
| Bt x ISO | 8 | 17.31 |
| Bt x ISO | 9 | 9.61 |
| Bt x ISO | 10 | 10.30 |
| Bt x ISO | 11 | 16.38 |
| Bt x ISO | 12 | 15.80 |
| Bt x ISO | 13 | 31.21 |
| Bt x ISO | 14 | 11.87 |
| Bt x ISO | 15 | 12.03 |
| Bt x ISO | 16 | 16.50 |
| Bt x ISO | 17 | 17.46 |
| Bt x ISO | 18 | 15.66 |
| Bt x ISO | 19 | 16.21 |
| Bt x ISO | 20 | 14.25 |
| Bt x ISO | 21 | 11.06 |
| Bt x ISO | 22 | 15.76 |
| Bt x ISO | 23 | 17.39 |
| Bt x ISO | 24 | 13.73 |
| Bt x ISO | 25 | 11.59 |
| Bt x ISO | 26 | 24.65 |
| Bt x ISO | 27 | 14.15 |
| Bt x ISO | 28 | 14.60 |
| Bt x ISO | 29 | 22.63 |
| (Bt OPV) x Bt | 1 | 15.74 |
| (Bt OPV) x Bt | 2 | 16.16 |
| (Bt OPV) x Bt | 3 | 43.71 |
| (Bt OPV) x Bt | 4 | 17.87 |
| (Bt OPV) x Bt | 5 | 27.17 |
| (Bt OPV) x Bt | 6 | 34.21 |
| (Bt OPV) x Bt | 7 | 17.54 |
| (Bt OPV) x Bt | 8 | 20.87 |
| (Bt OPV) x Bt | 9 | 21.96 |
| (Bt OPV) x Bt | 10 | 43.33 |
| (Bt OPV) x Bt | 11 | 16.23 |
| (Bt OPV) x Bt | 12 | 17.37 |
| (Bt OPV) x Bt | 13 | 41.06 |
| (Bt OPV) x Bt | 14 | 16.93 |
| (Bt OPV) x Bt | 15 | 7.33 |
| (Bt OPV) x Bt | 16 | 9.06 |
| (Bt OPV) x Bt | 17 | 10.45 |
| (Bt OPV) x Bt | 18 | 9.66 |
| (Bt OPV) x Bt | 19 | 10.19 |
| (Bt OPV) x Bt | 20 | 7.47 |
| (Bt OPV) x Bt | 21 | 11.96 |
| (Bt OPV) x Bt | 22 | 6.25 |
| (Bt OPV) x Bt | 23 | 18.70 |
| (Bt OPV) x Bt | 24 | 6.12 |
| (Bt OPV) x Bt | 25 | 9.06 |
| (Bt OPV) x OPV | 1 | 13.77 |
| (Bt OPV) x OPV | 2 | 13.18 |
| (Bt OPV) x OPV | 3 | 16.48 |
| (Bt OPV) x OPV | 4 | 11.84 |
| (Bt OPV) x OPV | 5 | 15.12 |
| (Bt OPV) x OPV | 6 | 15.74 |
| (Bt OPV) x OPV | 7 | 16.84 |
| (Bt OPV) x OPV | 8 | 4.61 |
| (Bt OPV) x OPV | 9 | 6.27 |
| (Bt OPV) x OPV | 10 | 8.57 |
| (Bt OPV) x OPV | 11 | 17.00 |
| (Bt OPV) x OPV | 12 | 13.19 |
| (Bt OPV) x OPV | 13 | 17.60 |
| (Bt OPV) x OPV | 14 | 16.28 |
| (Bt OPV) x OPV | 15 | 11.20 |
| (Bt OPV) x OPV | 16 | 9.10 |
| (Bt OPV) x OPV | 17 | 27.34 |
| (Bt OPV) x OPV | 18 | 23.63 |
| (Bt OPV) x OPV | 19 | 16.45 |
| (Bt OPV) x OPV | 20 | 23.29 |
| (Bt OPV) x OPV | 21 | 12.12 |
| (Bt OPV) x OPV | 22 | 14.98 |
| (Bt OPV) x OPV | 23 | 13.86 |
| (Bt OPV) x OPV | 24 | 10.39 |
| (Bt OPV) x OPV | 25 | 7.29 |
| (Bt OPV) x OPV | 26 | 15.61 |
| (Bt OPV) x OPV | 27 | 20.40 |
| (Bt OPV) x OPV | 28 | 18.35 |
| (Bt OPV) x OPV | 29 | 18.89 |
| (Bt OPV) x OPV | 30 | 18.12 |
| (Bt x ISO) x Bt | 1 | 14.15 |
| (Bt x ISO) x Bt | 2 | 22.99 |
| (Bt x ISO) x Bt | 3 | 28.36 |
| (Bt x ISO) x Bt | 4 | 24.30 |
| (Bt x ISO) x Bt | 5 | 18.90 |
| (Bt x ISO) x Bt | 6 | 29.94 |
| (Bt x ISO) x Bt | 7 | 32.18 |
| (Bt x ISO) x Bt | 8 | 15.90 |
| (Bt x ISO) x Bt | 9 | 40.06 |
| (Bt x ISO) x Bt | 10 | 39.13 |
| (Bt x ISO) x Bt | 11 | 24.24 |
| (Bt x ISO) x Bt | 12 | 22.20 |
| (Bt x ISO) x Bt | 13 | 15.48 |
| (Bt x ISO) x Bt | 14 | 23.38 |
| (Bt x ISO) x Bt | 15 | 29.16 |
| (Bt x ISO) x Bt | 16 | 21.10 |
| (Bt x ISO) x Bt | 17 | 19.56 |
| (Bt x ISO) x Bt | 18 | 30.55 |
| (Bt x ISO) x Bt | 19 | 28.78 |
| (Bt x ISO) x Bt | 20 | 38.98 |
| (Bt x ISO) x Bt | 21 | 11.40 |
| (Bt x ISO) x Bt | 22 | 21.75 |
| (Bt x ISO) x Bt | 23 | 35.70 |
| (Bt x ISO) x Bt | 24 | 28.95 |
| (Bt x ISO) x Bt | 25 | 12.00 |
| (Bt x ISO) x Bt | 26 | 16.42 |
| (Bt x ISO) x Bt | 27 | 18.10 |
| (Bt x ISO) x Bt | 28 | 12.68 |
| (Bt x ISO) x Bt | 29 | 20.23 |
| (Bt x ISO) x ISO | 1 | 21.64 |
| (Bt x ISO) x ISO | 2 | 17.02 |
| (Bt x ISO) x ISO | 3 | 25.48 |
| (Bt x ISO) x ISO | 4 | 22.63 |
| (Bt x ISO) x ISO | 5 | 25.52 |
| (Bt x ISO) x ISO | 6 | 14.92 |
| (Bt x ISO) x ISO | 7 | 12.18 |
| (Bt x ISO) x ISO | 8 | 24.41 |
| (Bt x ISO) x ISO | 9 | 28.40 |
| (Bt x ISO) x ISO | 10 | 20.21 |
| (Bt x ISO) x ISO | 11 | 18.87 |
| (Bt x ISO) x ISO | 12 | 18.36 |
| (Bt x ISO) x ISO | 13 | 17.37 |
| (Bt x ISO) x ISO | 14 | 27.64 |
| (Bt x ISO) x ISO | 15 | 10.04 |
| (Bt x ISO) x ISO | 16 | 16.83 |
| (Bt x ISO) x ISO | 17 | 15.93 |
| (Bt x ISO) x ISO | 18 | 14.80 |
| (Bt x ISO) x ISO | 19 | 19.92 |
| (Bt x ISO) x ISO | 20 | 20.18 |
| (Bt x ISO) x ISO | 21 | 12.19 |
| (Bt x ISO) x ISO | 22 | 10.67 |
| (Bt x ISO) x ISO | 23 | 30.47 |
| (Bt x ISO) x ISO | 24 | 16.15 |
| (Bt x ISO) x ISO | 25 | 16.10 |
| (Bt x ISO) x ISO | 26 | 16.64 |
| (Bt x ISO) x ISO | 27 | 20.81 |
| (Bt x ISO) x ISO | 28 | 13.79 |
| (Bt x OPV) x (Bt x OPV) | 1 | 18.05 |
| (Bt x OPV) x (Bt x OPV) | 2 | 11.42 |
| (Bt x OPV) x (Bt x OPV) | 3 | 22.48 |
| (Bt x OPV) x (Bt x OPV) | 4 | 18.13 |
| (Bt x OPV) x (Bt x OPV) | 5 | 25.39 |
| (Bt x OPV) x (Bt x OPV) | 6 | 33.72 |
| (Bt x OPV) x (Bt x OPV) | 7 | 13.59 |
| (Bt x OPV) x (Bt x OPV) | 8 | 15.05 |
| (Bt x OPV) x (Bt x OPV) | 9 | 20.31 |
| (Bt x OPV) x (Bt x OPV) | 10 | 35.13 |
| (Bt x OPV) x (Bt x OPV) | 11 | 18.34 |
| (Bt x OPV) x (Bt x OPV) | 12 | 21.40 |
| (Bt x OPV) x (Bt x OPV) | 13 | 18.34 |
| (Bt x OPV) x (Bt x OPV) | 14 | 20.88 |
| (Bt x OPV) x (Bt x OPV) | 15 | 24.67 |
| (Bt x OPV) x (Bt x OPV) | 16 | 10.53 |
| (Bt x OPV) x (Bt x OPV) | 17 | 13.07 |
| (Bt x OPV) x (Bt x OPV) | 18 | 14.64 |
| (Bt x OPV) x (Bt x OPV) | 19 | 22.02 |
| (Bt x OPV) x (Bt x OPV) | 20 | 19.19 |
| (Bt x OPV) x (Bt x OPV) | 21 | 12.41 |
| (Bt x OPV) x (Bt x OPV) | 22 | 27.31 |
| (Bt x OPV) x (Bt x OPV) | 23 | 12.25 |
| (Bt x OPV) x (Bt x OPV) | 24 | 26.21 |
| (Bt x OPV) x (Bt x OPV) | 25 | 20.89 |
| (Bt x OPV) x (Bt x OPV) | 26 | 21.90 |
| (Bt x OPV) x (Bt x OPV) | 27 | 14.72 |
| (Bt x ISO) x (Bt x ISO) | 1 | 28.83 |
| (Bt x ISO) x (Bt x ISO) | 2 | 30.97 |
| (Bt x ISO) x (Bt x ISO) | 3 | 24.45 |
| (Bt x ISO) x (Bt x ISO) | 4 | 12.77 |
| (Bt x ISO) x (Bt x ISO) | 5 | 22.35 |
| (Bt x ISO) x (Bt x ISO) | 6 | 11.83 |
| (Bt x ISO) x (Bt x ISO) | 7 | 19.41 |
| (Bt x ISO) x (Bt x ISO) | 8 | 26.26 |
| (Bt x ISO) x (Bt x ISO) | 9 | 23.60 |
| (Bt x ISO) x (Bt x ISO) | 10 | 30.17 |
| (Bt x ISO) x (Bt x ISO) | 11 | 48.55 |
| (Bt x ISO) x (Bt x ISO) | 12 | 25.13 |
| (Bt x ISO) x (Bt x ISO) | 13 | 30.59 |
| (Bt x ISO) x (Bt x ISO) | 14 | 30.01 |
| (Bt x ISO) x (Bt x ISO) | 15 | 20.16 |
| (Bt x ISO) x (Bt x ISO) | 16 | 12.65 |
| (Bt x ISO) x (Bt x ISO) | 17 | 22.01 |
| (Bt x ISO) x (Bt x ISO) | 18 | 32.79 |
| (Bt x ISO) x (Bt x ISO) | 19 | 5.16 |
| (Bt x ISO) x (Bt x ISO) | 20 | 13.63 |
| (Bt x ISO) x (Bt x ISO) | 21 | 39.97 |
| (Bt x ISO) x (Bt x ISO) | 22 | 16.27 |
| (Bt x ISO) x (Bt x ISO) | 23 | 36.89 |
| (Bt x ISO) x (Bt x ISO) | 24 | 14.92 |
| (Bt x ISO) x (Bt x ISO) | 25 | 13.67 |
| (Bt x ISO) x (Bt x ISO) | 26 | 15.90 |
| (Bt x ISO) x (Bt x ISO) | 27 | 16.03 |
| (Bt x ISO) x (Bt x ISO) | 28 | 24.19 |
| (Bt x ISO) x (Bt x ISO) | 29 | 20.87 |
