# Supplementary material for: Introgression of a cry1Ab transgene into open pollinated maize and its effect on Cry protein concentration and target pest survival
Source: PLoS One. 2019 Dec 16;14(12):e0226476. doi: 10.1371/journal.pone.0226476 (PMC6914330; doi:10.1371/journal.pone.0226476)
Supplement: S2 Table — (DOCX) [file pone.0226476.s002.docx]

**Table S2**

| **Maize treatment** | **Container number (5 larvae/container)** | **% Larval survival** | |
| --- | --- | --- | --- |
|  |  | **7 days** | **14 days** |
| Bt | 1 | 0 | 0 |
| Bt | 2 | 0 | 0 |
| Bt | 3 | 20 | 20 |
| Bt | 4 | 0 | 0 |
| Bt | 5 | 0 | 0 |
| Bt | 6 | 0 | 0 |
| Bt | 7 | 0 | 0 |
| Bt | 8 | 0 | 0 |
| Bt | 9 | 0 | 0 |
| Bt | 10 | 20 | 0 |
| Bt | 11 | 20 | 20 |
| Bt | 12 | 0 | 0 |
| Bt | 13 | 0 | 0 |
| Bt | 14 | 20 | 0 |
| Bt | 15 | 0 | 0 |
| Bt | 16 | 20 | 20 |
| Bt | 17 | 20 | 20 |
| Bt | 18 | 20 | 0 |
| Bt | 19 | 0 | 0 |
| Bt | 20 | 20 | 20 |
| Bt | 21 | 20 | 20 |
| Bt | 22 | 20 | 20 |
| Bt | 23 | 0 | 0 |
| Bt | 24 | 0 | 0 |
| Bt | 25 | 0 | 0 |
| Bt | 26 | 20 | 0 |
| Bt | 27 | 0 | 0 |
| Bt | 28 | 20 | 20 |
| Bt | 29 | 40 | 20 |
| Bt | 30 | 0 | 0 |
| OPV | 1 | 60 | 20 |
| OPV | 2 | 100 | 80 |
| OPV | 3 | 60 | 60 |
| OPV | 4 | 60 | 20 |
| OPV | 5 | 60 | 40 |
| OPV | 6 | 60 | 20 |
| OPV | 7 | 100 | 100 |
| OPV | 8 | 80 | 80 |
| OPV | 9 | 80 | 60 |
| OPV | 10 | 40 | 40 |
| OPV | 11 | 60 | 60 |
| OPV | 12 | 100 | 80 |
| OPV | 13 | 80 | 60 |
| OPV | 14 | 80 | 60 |
| OPV | 15 | 100 | 60 |
| OPV | 16 | 100 | 40 |
| OPV | 17 | 100 | 40 |
| OPV | 18 | 80 | 80 |
| OPV | 19 | 60 | 40 |
| OPV | 20 | 100 | 40 |
| OPV | 21 | 40 | 20 |
| OPV | 22 | 100 | 60 |
| OPV | 23 | 60 | 0 |
| OPV | 24 | 60 | 40 |
| OPV | 25 | 100 | 80 |
| OPV | 26 | 100 | 60 |
| OPV | 27 | 80 | 80 |
| OPV | 28 | 100 | 60 |
| OPV | 29 | 20 | 20 |
| OPV | 30 | 100 | 100 |
| ISO | 1 | 100 | 100 |
| ISO | 2 | 40 | 60 |
| ISO | 3 | 60 | 40 |
| ISO | 4 | 80 | 40 |
| ISO | 5 | 20 | 0 |
| ISO | 6 | 60 | 60 |
| ISO | 7 | 60 | 40 |
| ISO | 8 | 100 | 40 |
| ISO | 9 | 60 | 20 |
| ISO | 10 | 60 | 40 |
| ISO | 11 | 60 | 60 |
| ISO | 12 | 80 | 60 |
| ISO | 13 | 0 | 0 |
| ISO | 14 | 60 | 80 |
| ISO | 15 | 60 | 20 |
| ISO | 16 | 60 | 20 |
| ISO | 17 | 40 | 40 |
| ISO | 18 | 60 | 60 |
| ISO | 19 | 60 | 20 |
| ISO | 20 | 80 | 40 |
| ISO | 21 | 80 | 60 |
| ISO | 22 | 60 | 60 |
| ISO | 23 | 60 | 60 |
| ISO | 24 | 60 | 60 |
| ISO | 25 | 60 | 60 |
| ISO | 26 | 20 | 0 |
| ISO | 27 | 80 | 80 |
| ISO | 28 | 80 | 60 |
| ISO | 29 | 80 | 40 |
| ISO | 30 | 100 | 80 |
| Bt x OPV | 1 | 20 | 20 |
| Bt x OPV | 2 | 40 | 20 |
| Bt x OPV | 3 | 20 | 0 |
| Bt x OPV | 4 | 20 | 20 |
| Bt x OPV | 5 | 20 | 0 |
| Bt x OPV | 6 | 20 | 20 |
| Bt x OPV | 7 | 20 | 0 |
| Bt x OPV | 8 | 0 | 0 |
| Bt x OPV | 9 | 40 | 20 |
| Bt x OPV | 10 | 40 | 0 |
| Bt x OPV | 11 | 40 | 20 |
| Bt x OPV | 12 | 60 | 60 |
| Bt x OPV | 13 | 20 | 20 |
| Bt x OPV | 14 | 20 | 20 |
| Bt x OPV | 15 | 20 | 20 |
| Bt x OPV | 16 | 0 | 0 |
| Bt x OPV | 17 | 40 | 20 |
| Bt x OPV | 18 | 40 | 20 |
| Bt x OPV | 19 | 20 | 20 |
| Bt x OPV | 20 | 0 | 0 |
| Bt x OPV | 21 | 40 | 0 |
| Bt x OPV | 22 | 0 | 0 |
| Bt x OPV | 23 | 20 | 0 |
| Bt x OPV | 24 | 0 | 0 |
| Bt x OPV | 25 | 40 | 40 |
| Bt x ISO | 1 | 0 | 0 |
| Bt x ISO | 2 | 0 | 0 |
| Bt x ISO | 3 | 60 | 20 |
| Bt x ISO | 4 | 0 | 0 |
| Bt x ISO | 5 | 0 | 0 |
| Bt x ISO | 6 | 40 | 40 |
| Bt x ISO | 7 | 0 | 0 |
| Bt x ISO | 8 | 20 | 0 |
| Bt x ISO | 9 | 20 | 20 |
| Bt x ISO | 10 | 0 | 0 |
| Bt x ISO | 11 | 20 | 20 |
| Bt x ISO | 12 | 20 | 20 |
| Bt x ISO | 13 | 20 | 20 |
| Bt x ISO | 14 | 20 | 20 |
| Bt x ISO | 15 | 40 | 20 |
| Bt x ISO | 16 | 0 | 0 |
| Bt x ISO | 17 | 20 | 0 |
| Bt x ISO | 18 | 0 | 0 |
| Bt x ISO | 19 | 20 | 20 |
| Bt x ISO | 20 | 20 | 20 |
| Bt x ISO | 21 | 20 | 0 |
| Bt x ISO | 22 | 20 | 0 |
| Bt x ISO | 23 | 20 | 20 |
| Bt x ISO | 24 | 0 | 0 |
| Bt x ISO | 25 | 40 | 20 |
| Bt x ISO | 26 | 0 | 0 |
| (Bt x OPV) x Bt | 1 | 0 | 0 |
| (Bt x OPV) x Bt | 2 | 80 | 40 |
| (Bt x OPV) x Bt | 3 | 0 | 0 |
| (Bt x OPV) x Bt | 4 | 0 | 0 |
| (Bt x OPV) x Bt | 5 | 20 | 20 |
| (Bt x OPV) x Bt | 6 | 0 | 0 |
| (Bt x OPV) x Bt | 7 | 0 | 0 |
| (Bt x OPV) x Bt | 8 | 40 | 20 |
| (Bt x OPV) x Bt | 9 | 60 | 20 |
| (Bt x OPV) x Bt | 10 | 0 | 0 |
| (Bt x OPV) x Bt | 11 | 40 | 20 |
| (Bt x OPV) x Bt | 12 | 40 | 40 |
| (Bt x OPV) x Bt | 13 | 20 | 0 |
| (Bt x OPV) x Bt | 14 | 0 | 0 |
| (Bt x OPV) x Bt | 15 | 20 | 0 |
| (Bt x OPV) x Bt | 16 | 0 | 0 |
| (Bt x OPV) x Bt | 17 | 40 | 40 |
| (Bt x OPV) x Bt | 18 | 20 | 40 |
| (Bt x OPV) x Bt | 19 | 0 | 0 |
| (Bt x OPV) x Bt | 20 | 20 | 0 |
| (Bt x OPV) x Bt | 21 | 20 | 0 |
| (Bt x OPV) x Bt | 22 | 0 | 0 |
| (Bt x OPV) x OPV | 1 | 20 | 20 |
| (Bt x OPV) x OPV | 2 | 20 | 20 |
| (Bt x OPV) x OPV | 3 | 40 | 20 |
| (Bt x OPV) x OPV | 4 | 0 | 0 |
| (Bt x OPV) x OPV | 5 | 0 | 0 |
| (Bt x OPV) x OPV | 6 | 0 | 0 |
| (Bt x OPV) x OPV | 7 | 0 | 0 |
| (Bt x OPV) x OPV | 8 | 20 | 20 |
| (Bt x OPV) x OPV | 9 | 0 | 0 |
| (Bt x OPV) x OPV | 10 | 20 | 0 |
| (Bt x OPV) x OPV | 11 | 20 | 20 |
| (Bt x OPV) x OPV | 12 | 0 | 0 |
| (Bt x OPV) x OPV | 13 | 20 | 20 |
| (Bt x OPV) x OPV | 14 | 40 | 0 |
| (Bt x OPV) x OPV | 15 | 20 | 20 |
| (Bt x OPV) x OPV | 16 | 20 | 0 |
| (Bt x OPV) x OPV | 17 | 0 | 0 |
| (Bt x OPV) x OPV | 18 | 80 | 60 |
| (Bt x OPV) x OPV | 19 | 0 | 0 |
| (Bt x OPV) x OPV | 20 | 20 | 20 |
| (Bt x OPV) x OPV | 21 | 40 | 20 |
| (Bt x OPV) x OPV | 22 | 0 | 0 |
| (Bt x OPV) x OPV | 23 | 20 | 20 |
| (Bt x OPV) x OPV | 24 | 0 | 0 |
| (Bt x OPV) x OPV | 25 | 0 | 0 |
| (Bt x OPV) x OPV | 26 | 40 | 40 |
| (Bt x OPV) x OPV | 27 | 0 | 0 |
| (Bt x OPV) x OPV | 28 | 40 | 20 |
| (Bt x ISO) x Bt | 1 | 0 | 0 |
| (Bt x ISO) x Bt | 2 | 40 | 40 |
| (Bt x ISO) x Bt | 3 | 20 | 20 |
| (Bt x ISO) x Bt | 4 | 0 | 0 |
| (Bt x ISO) x Bt | 5 | 40 | 40 |
| (Bt x ISO) x Bt | 6 | 0 | 0 |
| (Bt x ISO) x Bt | 7 | 0 | 0 |
| (Bt x ISO) x Bt | 8 | 40 | 20 |
| (Bt x ISO) x Bt | 9 | 40 | 40 |
| (Bt x ISO) x Bt | 10 | 20 | 20 |
| (Bt x ISO) x Bt | 11 | 0 | 0 |
| (Bt x ISO) x Bt | 12 | 0 | 0 |
| (Bt x ISO) x Bt | 13 | 40 | 20 |
| (Bt x ISO) x Bt | 14 | 0 | 0 |
| (Bt x ISO) x Bt | 15 | 40 | 40 |
| (Bt x ISO) x Bt | 16 | 0 | 0 |
| (Bt x ISO) x Bt | 17 | 20 | 40 |
| (Bt x ISO) x Bt | 18 | 0 | 0 |
| (Bt x ISO) x Bt | 19 | 20 | 0 |
| (Bt x ISO) x Bt | 20 | 40 | 40 |
| (Bt x ISO) x Bt | 21 | 0 | 0 |
| (Bt x ISO) x Bt | 22 | 0 | 0 |
| (Bt x ISO) x Bt | 23 | 20 | 20 |
| (Bt x ISO) x Bt | 24 | 20 | 20 |
| (Bt x ISO) x Bt | 25 | 20 | 20 |
| (Bt x ISO) x Bt | 26 | 0 | 0 |
| (Bt x ISO) x ISO | 1 | 0 | 0 |
| (Bt x ISO) x ISO | 2 | 40 | 20 |
| (Bt x ISO) x ISO | 3 | 0 | 0 |
| (Bt x ISO) x ISO | 4 | 20 | 0 |
| (Bt x ISO) x ISO | 5 | 20 | 20 |
| (Bt x ISO) x ISO | 6 | 20 | 20 |
| (Bt x ISO) x ISO | 7 | 0 | 0 |
| (Bt x ISO) x ISO | 8 | 0 | 0 |
| (Bt x ISO) x ISO | 9 | 20 | 0 |
| (Bt x ISO) x ISO | 10 | 0 | 0 |
| (Bt x ISO) x ISO | 11 | 20 | 0 |
| (Bt x ISO) x ISO | 12 | 40 | 40 |
| (Bt x ISO) x ISO | 13 | 20 | 0 |
| (Bt x ISO) x ISO | 14 | 0 | 0 |
| (Bt x ISO) x ISO | 15 | 0 | 0 |
| (Bt x ISO) x ISO | 16 | 20 | 0 |
| (Bt x ISO) x ISO | 17 | 0 | 0 |
| (Bt x ISO) x ISO | 18 | 0 | 0 |
| (Bt x ISO) x ISO | 19 | 60 | 40 |
| (Bt x ISO) x ISO | 20 | 40 | 20 |
| (Bt x ISO) x ISO | 21 | 0 | 0 |
| (Bt x ISO) x ISO | 22 | 40 | 40 |
| (Bt x ISO) x ISO | 23 | 20 | 0 |
| (Bt x ISO) x ISO | 24 | 60 | 20 |
| (Bt x ISO) x ISO | 25 | 0 | 0 |
| (Bt x ISO) x ISO | 26 | 0 | 0 |
| (Bt x ISO) x ISO | 27 | 20 | 20 |
| (Bt x ISO) x ISO | 28 | 0 | 0 |
| (Bt x OPV) x (Bt x OPV) | 1 | 0 | 0 |
| (Bt x OPV) x (Bt x OPV) | 2 | 20 | 0 |
| (Bt x OPV) x (Bt x OPV) | 3 | 0 | 0 |
| (Bt x OPV) x (Bt x OPV) | 4 | 60 | 20 |
| (Bt x OPV) x (Bt x OPV) | 5 | 0 | 0 |
| (Bt x OPV) x (Bt x OPV) | 6 | 40 | 0 |
| (Bt x OPV) x (Bt x OPV) | 7 | 20 | 20 |
| (Bt x OPV) x (Bt x OPV) | 8 | 20 | 0 |
| (Bt x OPV) x (Bt x OPV) | 9 | 0 | 0 |
| (Bt x OPV) x (Bt x OPV) | 10 | 20 | 0 |
| (Bt x OPV) x (Bt x OPV) | 11 | 40 | 20 |
| (Bt x OPV) x (Bt x OPV) | 12 | 40 | 40 |
| (Bt x OPV) x (Bt x OPV) | 13 | 0 | 0 |
| (Bt x OPV) x (Bt x OPV) | 14 | 0 | 20 |
| (Bt x OPV) x (Bt x OPV) | 15 | 0 | 0 |
| (Bt x OPV) x (Bt x OPV) | 16 | 0 | 0 |
| (Bt x OPV) x (Bt x OPV) | 17 | 40 | 20 |
| (Bt x OPV) x (Bt x OPV) | 18 | 60 | 20 |
| (Bt x OPV) x (Bt x OPV) | 19 | 40 | 20 |
| (Bt x OPV) x (Bt x OPV) | 20 | 0 | 0 |
| (Bt x OPV) x (Bt x OPV) | 21 | 20 | 0 |
| (Bt x OPV) x (Bt x OPV) | 22 | 60 | 20 |
| (Bt x OPV) x (Bt x OPV) | 23 | 0 | 0 |
| (Bt x OPV) x (Bt x OPV) | 24 | 0 | 0 |
| (Bt x OPV) x (Bt x OPV) | 25 | 0 | 0 |
| (Bt x OPV) x (Bt x OPV) | 26 | 20 | 20 |
| (Bt x OPV) x (Bt x OPV) | 27 | 0 | 0 |
| (Bt x OPV) x (Bt x OPV) | 28 | 40 | 40 |
| (Bt x ISO) x (Bt x ISO) | 1 | 20 | 20 |
| (Bt x ISO) x (Bt x ISO) | 2 | 40 | 0 |
| (Bt x ISO) x (Bt x ISO) | 3 | 0 | 0 |
| (Bt x ISO) x (Bt x ISO) | 4 | 20 | 0 |
| (Bt x ISO) x (Bt x ISO) | 5 | 0 | 0 |
| (Bt x ISO) x (Bt x ISO) | 6 | 20 | 20 |
| (Bt x ISO) x (Bt x ISO) | 7 | 20 | 0 |
| (Bt x ISO) x (Bt x ISO) | 8 | 40 | 0 |
| (Bt x ISO) x (Bt x ISO) | 9 | 20 | 20 |
| (Bt x ISO) x (Bt x ISO) | 10 | 0 | 0 |
| (Bt x ISO) x (Bt x ISO) | 11 | 40 | 20 |
| (Bt x ISO) x (Bt x ISO) | 12 | 0 | 0 |
| (Bt x ISO) x (Bt x ISO) | 13 | 40 | 0 |
| (Bt x ISO) x (Bt x ISO) | 14 | 0 | 0 |
| (Bt x ISO) x (Bt x ISO) | 15 | 0 | 0 |
| (Bt x ISO) x (Bt x ISO) | 16 | 0 | 0 |
| (Bt x ISO) x (Bt x ISO) | 17 | 40 | 20 |
| (Bt x ISO) x (Bt x ISO) | 18 | 0 | 0 |
| (Bt x ISO) x (Bt x ISO) | 19 | 0 | 0 |
| (Bt x ISO) x (Bt x ISO) | 20 | 20 | 0 |
| (Bt x ISO) x (Bt x ISO) | 21 | 0 | 0 |
| (Bt x ISO) x (Bt x ISO) | 22 | 0 | 0 |
| (Bt x ISO) x (Bt x ISO) | 23 | 20 | 20 |
| (Bt x ISO) x (Bt x ISO) | 24 | 0 | 0 |
| (Bt x ISO) x (Bt x ISO) | 25 | 20 | 0 |
| (Bt x ISO) x (Bt x ISO) | 26 | 20 | 0 |
| (Bt x ISO) x (Bt x ISO) | 27 | 0 | 0 |
| (Bt x ISO) x (Bt x ISO) | 28 | 0 | 0 |
| (Bt x ISO) x (Bt x ISO) | 29 | 20 | 0 |
| (Bt x ISO) x (Bt x ISO) | 30 | 40 | 40 |
